# Supplementary material for: Patterns of Intron Gain and Loss in Fungi
Source: PLoS Biol. 2004 Nov 30;2(12):e422. doi: 10.1371/journal.pbio.0020422 (PMC532390; doi:10.1371/journal.pbio.0020422)
Supplement: Table S1 — Also available at http://genes.mit.edu/NielsenEtAl/. (4.3 MB ZIP). [file pbio.0020422.st001.zip › NielsenEtAl/html/573.html]

AN8820.1.NCU03804.1.MG07456.2.FG06103.1


```
 CLUSTAL W (1.82) Multiple Sequence Alignments - Introns Inserted


Sequence 1: NCU03804.1	517 aa
Sequence 2: MG07456.2	535 aa
Sequence 3: FG06103.1	514 aa
Sequence 4: AN8820.1	525 aa
Alignment Length: 543 aa
Number Identitical Residues: 412 aa
Alignment Score (without introns) 17600


MG07456.2 	MEDGTQVNTAERVCKD1VQAPAMFKPTDEQFFEDESHEKPDIAFLKQHFYREGRLTHEQA
NCU03804.1	MEDGSQVSTMERVCKD1VQAPAMFKPSDEQFFEDETHTKPDIQFLKQHFYREGRLTEEQA
FG06103.1 	MEDNTQVSTMERVCKD1VQAPAMYKPTDEQFFEDETHSKPNLQFLKQHFYREGRLTEEQA
AN8820.1  	MEDGTQVSTLERVVKE1VQAPALNKPSDDQFWDPEEPTKPNLQFLKQHFYREGRLTEDQA
          	***.:**.* *** *: *****: **:*:**:: *   **:: *************.:**

MG07456.2 	LWILKKGTEILRSEPNLLEMDAPITVCGDVHGQYYDLMKLFEVGGDPAETRYLFLGDYVD
NCU03804.1	LWIIREGTKLLRAEPNLLEMDAPITVCGDVHGQYYDLMKLFEVGGDPAETRYLFLGDYVD
FG06103.1 	LWILKKGTELLRAEPNLLEMDAPITVCGDVHGQYYDLMKLFEVGGDPSETRYLFLGDYVD
AN8820.1  	LWIIQAGTQILKSEPNLLEMDAPITVCGDVHGQYYDLMKLFEVGGDPAETRYLFLGDYVD
          	***:: **::*::**********************************:************

MG07456.2 	RGYFSIE~CVLYLWCLKIHYPKTLWLLRGNHECRHLTDYFTFKLECKHKYSEAVYDACME
NCU03804.1	RGYFSIE~CVLYLWALKIHYPKTLWLLRGNHECRHLTDYFTFKLECKHKYSEAIYEACME
FG06103.1 	RGYFSIE~CVLYLWCLKIHYPKTLWLLRGNHECRHLTDYFTFKLECKHKYSETIYEACMD
AN8820.1  	RGYFSIE0CVLYLWALKIWYPNTLWLLRGNHECRHLTDYFTFKLECKHKYSERIYEACIE
          	******* ******.*** **:****************************** :*:**::

MG07456.2 	SFCSLPLAAVMNKQFLCIHGGLSPELHTLDDIRN0IDRFREPPTQGLMCDILWADPLEDF
NCU03804.1	SFCCLPLAAVMNKQFLCIHGGLSPELHTLDDIRN0IDRFREPPTQGLMCDILWADPLEDF
FG06103.1 	SFCALPLAAVMNKQFLCIHGGLSPELHTLDDLKS0IDRFREPPTQGLMCDILWADPLEDF
AN8820.1  	SFCALPLAAVMNKQFLCIHGGLSPELHTLEDIKS0IDRFREPPTHGLMCDILWADPLEDF
          	***.*************************:*::. *********:***************

MG07456.2 	GQEKTSDFFLHNHVRGCSYFFSYPAACAFLEKNNLLSVIRAHEAQDAGYRMYRKTRTTGF
NCU03804.1	GQEKTTDFFVHNHVRGCSYFFSYSAACHFLEKNNLLSIIRAHEAQDAGYRMYRKTRTTGF
FG06103.1 	GQEKTSDYFLHNHVRGCSYFFSYPAACAFLEKNNLLSIIRAHEAQDAGYRMYRKTRTTGF
AN8820.1  	GQEKTGDYFIHNSVRGCSYFFSYPAACAFLEKNNLLSVIRAHEAQDAGYRMYRKTRTTGF
          	***** *:*:** **********.*** *********:**********************

MG07456.2 	PSVMTIFSAPNYLDVYNNKAAVLKYENNVMNIRQFNCTPHPYWLPNFMDVFTWSLPFVGE
NCU03804.1	PSVMTIFSAPNYLDVYNNKAAVLKYENNVMNIRQFNCTPHPYWLPNFMDVFTWSLPFVGE
FG06103.1 	PSVMTIFSAPNYLDVYNNKAAVLKYENNVMNIRQFNCTPHPYWLPNFMDVFTWSLPFVGE
AN8820.1  	PSVMTIFSAPNYLDVYNNKAAVLKYENNVMNIRQFNCTPHPYWLPNFMDVFTWSLPFVGE
          	************************************************************

MG07456.2 	KITDMLLAILGTCSEEELREGDSSSTSPGPQSPPQTPALPEAAGDGEGSSTSANAAAMDP
NCU03804.1	KITDMLIAILSTCSEEELRE-DSATTSPGSASP----ALPSAANQ-------------DP
FG06103.1 	KITDMLIAILSTCSEEELKEETPSSTSPGPASP----ALSN-----------------DP
AN8820.1  	KITDMLIAILNTCSKEELEDETPSTISPAEPSP----PMPMDT--------------VDT
          	******:***.***:***.:  .:: **.  **    .:.  :               *.

MG07456.2 	ESIEFKRRAIKNKILAIGRLSRVFQVLREESERVTELKTVSGGRLPAGTLMLGAEGIKNA
NCU03804.1	DSIEFKRRAIKNKILAIGRLSRVFQVLREESERVTELKTVSGGRLPAGTLMLGAEGIKNA
FG06103.1 	ESIEVRRRAIKNKILAIGRLSRVFQVLREESEKVTELKTVSGGRLPAGTLMLGAEGLKNA
AN8820.1  	ESTEFKRRAIKNKILAIGRLSRVFQVLREESERVTELKTAAGGRLPAGTLMLGAEGIKQA
          	:* *.:**************************:******.:***************:*:*

MG07456.2 	ISSFEDARKVDLQNERLPPSHEEVVRQNEEERAQALERATKEADNDKKLQTLSRRLST~-
NCU03804.1	ISSFEDARKVDLQNERLPPSHDEVVKMQDEERAQALERATREADNDKKLQTLSRRLST~-
FG06103.1 	INGFEDARKVDLQNERLPPTHDEVVKHQEEERNTALQKAAEDANNDTKLQQLSRRLST~-
AN8820.1  	ITNFEDARKVDLQNERLPPSHDEVVRRSEEERRIALDRAQHEADNDTGLATVARRISM2K
          	*..****************:*:***: .:***  **::* .:*:**. *  ::**:*  .

MG07456.2 	-------
NCU03804.1	-------
FG06103.1 	-------
AN8820.1  	IPSTTRR
          	 .:::
```
